# Reappraisal of the Prognostic Factors of Outcome and Recovery Time in Patients with Idiopathic Bell’s Palsy: A Retrospective Single-Center Analysis

**DOI:** 10.3390/jpm11030171

**Published:** 2021-03-02

**Authors:** Chi-Hao Peng, Jiun-Liang Chen, Ming-Feng Liao, Jung-Lung Hsu, Hui-Ching Hsu, Long-Sun Ro

**Affiliations:** 1 Division of Chinese Internal Medicine, Center for Traditional Chinese Medicine, Chang Gung Memorial Hospital, Taoyuan 333, Taiwan; toponchu@gmail.com (C.-H.P.); a12015@cgmh.org.tw (J.-L.C.); 2 School of Traditional Chinese Medicine, College of Medicine, Chang Gung University, Taoyuan 333, Taiwan; 3 Department of Neurology, Chang Gung Memorial Hospital, Taipei 105, Taiwan; mingfengliao@hotmail.com (M.-F.L.); tulu@ms36.hinet.net (J.-L.H.); 4 Department of Traditional Chinese Medicine, Division of Chinese Acupuncture and Traumatology, Chang Gung Memorial Hospital, Taipei 105, Taiwan; faithjanet@gmail.com

**Keywords:** Bell’s palsy, facial electroneurography, blink reflex test, prognosis, recovery time

## Abstract

Study Objectives: This retrospective study investigated prognostic factors and recovery time in patients with Bell’s palsy after different doses and durations of oral glucocorticoid treatments. Subjects and Methods: A total of 396 patients initially diagnosed with Bell’s palsy that had visited the Department of Neurology of Chang Gung Memorial Hospital, Taoyuan, a tertiary referral medical center in Taiwan, between January 2014 and December 2018 were included. Medical records, facial electroneurography (fENoG), and blink reflex (BR) tests were reviewed and analyzed. A favorable outcome was defined as patients who improved to grade ≤ II, and an unfavorable outcome was defined as patients who improved to grade ≥ III in 6 months according to the House–Brackmann (HB) grading system. Results: The rate of favorable outcomes was 89.4% (354 of 396 patients) at the 6-month follow-up. A favorable outcome (HB less than grade II) was associated with a delayed BR (odds ratio, OR, 5.38; 95% CI, 1.82 to 15.90) and fENoG values (the lesion side/the healthy side) over 33% (OR, 6.67; 95% CI, 3.02 to 14.71). The recovery time was significantly shorter for those with a delayed BR than for those with an absent BR and shorter for those with good fENoG values (>33%) than for those with poor values (≤33%). However, treatment without or with different doses and durations of oral glucocorticoid did not influence the final outcome or recovery time in this study. Conclusions: The fENoG and BR tests were significant and highly valuable examinations for predicting the final outcome. Moreover, age younger than 60 years, a delayed BR, and fENoG values > 33% were associated with shorter recovery times.

## 1. Introduction

Bell’s palsy is an acute unilateral facial paralysis with an unknown cause [1]. The prognosis of Bell’s palsy is generally good, with approximately 70% of patients recovering spontaneously by three to six months [2]. Some factors that have been associated with the recovery time from Bell’s palsy include age, diabetes, and pregnancy [3,4,5]. Electrophysiological tests such as facial electroneurography (fENoG) and blink reflex (BR) tests have been used to evaluate facial nerve function and to predict outcome [6,7]. Unfavorable fENoG values and an absent BR test are associated with a worse outcome in patients with Bell’s palsy [7].

The main treatment for facial palsy is short-term oral glucocorticoids [8]. A previous study showed that prednisolone therapy should be started within three days after onset to improve facial nerve function and decrease the recovery time [9]. However, the prognostic factors and recovery time of patients who need to receive prednisolone doses and the treatment duration are not well studied.

Thus, the aim of this study was to analyze potential variables associated with the outcomes and to investigate the association between electrophysiology test results and the recovery time in patients with Bell’s palsy after conservative treatment or with different doses and durations of oral glucocorticoids.

## 2. Materials and Methods

This retrospective study included patients who were diagnosed with Bell’s palsy (ICD-10: G510, ICD-9: 3510) and visited the Department of Neurology at Chang Gung Memorial Hospital, Taoyuan, Taiwan, from January 2014 to December 2018. Medical records of 1115 patients were reviewed. A total of 719 subjects were excluded for herpes zoster, Ramsay Hunt syndrome, recurrent Bell’s palsy, stroke history, malignancy, pregnancy, no specific onset time, or loss to follow-up. In total, 396 patients were included in the study. The study protocol was approved by the Research and Ethics Committee of Chang Gung Memorial Hospital (CGMH) in Taiwan (serial number: 201901622B0). The requirement for written informed consent was waived by CGMH due to the retrospective nature of this case research.

The baseline demographic and clinical variables were recorded, including age, sex, affected site, personal history (smoking, alcohol drinking, and betel nut chewing), and past medical history (diabetes, hypertension, and hyperlipidemia). Low-dose steroids were defined as ≤0.5 mg/kg prednisolone/day, and high-dose steroids were defined as >0.5 mg/kg prednisolone/day [10]. The improvement of facial nerve function was assessed based on the House–Brackmann (HB) grading system [11]. We defined favorable outcomes as patients who improved to HB grade ≤ II and unfavorable outcomes as patients who improved to HB grade ≥ III. The recovery time was defined from the onset time of facial palsy to a favorable outcome or an unfavorable outcome with stationary improvement without further treatment at the 6-month follow-up.

**Figure 1:**
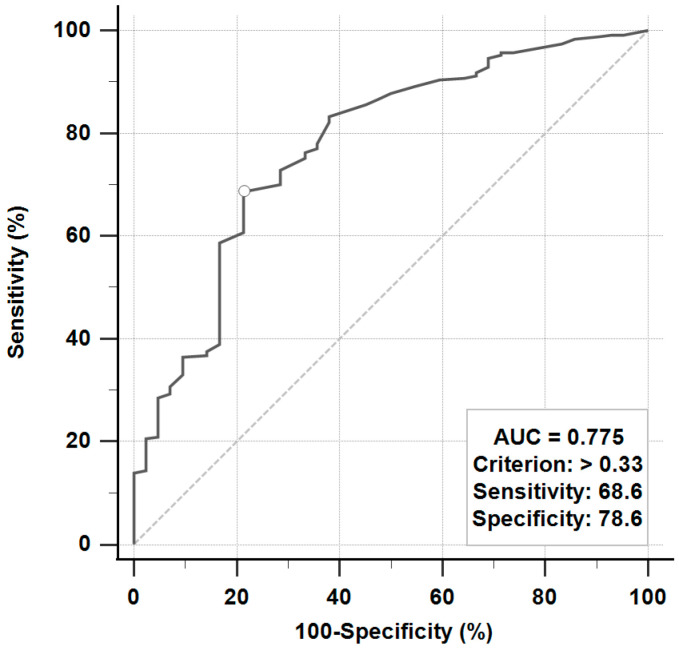
The receiver operating characteristic (ROC) curve to evaluate facial electroneurography for the prediction of favorable or unfavorable outcomes.

fENoG and BR tests to evaluate the prognosis of Bell’s palsy were performed from days 4 to 21 after the onset of facial weakness. The fENoG values were recorded as the amplitude of the lesion site divided by the healthy site. The receiver operating characteristic (ROC) curve was used to determine the cutoff point of favorable and unfavorable outcomes (Figure 1). A good fENoG value was defined as >33%, and a poor fENoG value was defined as ≤33%. In the BR test, the latency of ipsilateral R1, R2, and contralateral R2 were measured. The latency of the ipsilateral (the lesion) R1 > 12 ms and ipsilateral or contralateral R2 > 35 ms were classified as delayed, and one of the ipsilateral R1, R2, and contralateral R2 with no response was classified as absent [7].

Qualitative variables are presented as absolute numbers and percentages, while quantitative variables are expressed as the mean (95% CI). The demographic and clinical variables classified with favorable and unfavorable outcomes were compared using chi-square tests. Multivariable logistic regression analysis was used to evaluate favorable outcomes in patients with idiopathic Bell’s palsy. The associations were reported as odds ratios (ORs) with 95% CIs. An independent t-test was performed to analyze the different recovery times classified with fENoG measurements and BR tests. The clinical variables on the recovery time in patients with Bell’s palsy were analyzed by multivariable regression analysis. We defined age <60 as 1 and ≥60 as 0, delayed blink reflex as 1 and absent blink reflex as 0, and facial electroneurography value >33% as 1 and ≤33% as 0. All tests were two tailed, and a *p*-value < 0.05 was considered statistically significant. All statistical analyses were performed using software (version 19.6; MedCalc, Ostend, Belgium).

## 3. Results

A total of 396 patients first diagnosed with Bell’s palsy were included in the study (179 females, 45.2%, and 217 males, 54.8%) (Table 1). The mean (95% CI) age was 45.7 (44.1–47.3) years. Of the 396 subjects, 149 (37.6%) had a delayed BR, 247 (62.4%) had an absent BR, 254 (64.1%) had good fENoG values of >33%, and 142 (35.9%) had poor fENoG values of ≤33%. The rate of favorable outcomes was 89.4% (354 of 396 patients) at the 6-month follow-up. Among 354 patients with favorable outcomes, 283 (79.9%) were younger than 60 years, 209 (59%) had absent BR, and 244 (68.9%) had fENoG values over 33%. In addition, significant differences in favorable and unfavorable outcomes were found in different BR patterns (*p* = 0.0001) and fENoG values (*p* < 0.0001).

Multivariable logistic regression analysis was used to assess the independent factors associated with the outcome (Table 2). A favorable outcome (HB less than grade II) was associated with a delayed blink reflex (OR, 5.38; 95% CI, 1.82 to 15.90) and fENoG values over 33% (OR, 6.67; 95% CI, 3.02 to 14.71). The area under the ROC curve was 0.81 (95% CI, 0.77 to 0.85). However, different doses and durations of oral glucocorticoid treatments were not associated with favorable outcomes.

**Figure 2:**
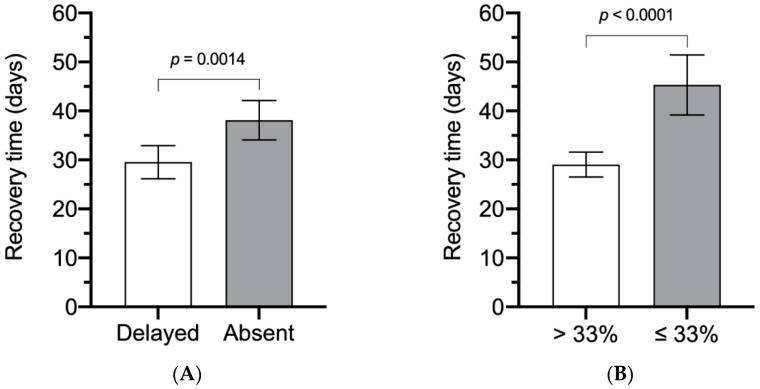
The recovery time classified with different blink reflex (BR) test and facial electroneurography (fENoG) values. (**A**) Different recovery times between patients with a delayed BR and those with an absent BR. (**B**) Different recovery times for those with fENoG values >33% and ≤33%.

When classified by different BR test and fENoG values (Figure 2), the recovery time was significantly shorter for those with a delayed BR (29.54 days, 95% CI, 26.15 to 32.93) than those with an absent BR (38.11 days, 95% CI, 34.10 to 42.13) (*p* = 0.0014) and shorter for those with fENoG values >33% (29.06 days, 95% CI, 26.50 to 31.63) than for those with values ≤33% (45.31 days, 95% CI, 39.20 to 51.42) (*p* < 0.0001).

Multivariable regression analysis was performed to evaluate the independent variables associated with the recovery time (Table 3). Age, BR test, and fENoG values were important independent factors in predicting the recovery time. Age younger than 60 years, delay-type BR test, and fENoG values >33% were associated with shorter recovery times.

## 4. Discussion

Our study was designed to evaluate the factors associated with a favorable outcome and to investigate the prognostic value of fENoG and BR tests in the recovery time of Bell’s palsy. In the present study, electrophysiological tests were associated with the outcome in patients with Bell’s palsy. However, the dosage and duration of glucocorticoid treatment did not influence the final outcome.

Previous studies have reported that age is a parameter that influences the final outcome. Peitersen showed that children (aged ≤ 14 years) had the most favorable prognosis, while older patients (aged > 60 years) had the worst outcomes [2]. Yoo et al. reported that age younger than 40 years was associated with a favorable outcome [12]. In our study, age less than 60 years was not associated with favorable outcomes. However, younger age may be associated with shorter recovery time in the study.

In a previous study, there was no significant difference in sex distribution among patients with Bell’s palsy. The full recovery rate of females (72%) was similar to that of males (69%) [2]. Yoo et al. showed that gender was not associated with a favorable outcome (OR, 1.04; 95% CI, 0.78 to 1.38) [12]. This finding was compatible with our study results showing that sex did not influence the final outcome or recovery time.

To our knowledge, the relationship between personal history (smoking, alcohol, and betel nut consumption) and the outcome of patients with Bell’s palsy has not been reported. Kim et al. reported that smoking did not show any relationship with the occurrence of Bell’s palsy [13]. In our study, free smoking, alcohol, and betel nut consumption was not associated with a favorable outcome.

Previous studies have reported that diabetes was significantly associated with unfavorable outcomes. Takemoto et al. showed that diabetes was a prognostic factor in Bell’s palsy [14]. Diabetes related to microangiopathy and neuropathy may result in poor recovery in patients with facial palsy [15]. However, our findings only demonstrated that the absence of diabetes had a higher odds ratio (or a tendency) for favorable outcomes than the presence of diabetes, but the data did not reach statistical significance.

Yeo et al. reported that hypertension was not a prognostic factor in patients with Bell’s palsy [16]. Similar to our study, the presence of hypertension was not a good prognostic factor for favorable outcomes. In contrast, Lee et al. showed that the final recovery rate of Bell’s palsy was influenced by accompanying hypertension [3]. Yoo et al. further investigated whether controlled hypertension was associated with a good outcome [12]. The prognostic value in the presence of hypertension seems to be controversial. Hypertension or controlled medication may influence the recovery of Bell’s palsy.

Several studies have shown the prognostic value of fENoG and BR tests [6,7]. However, the association between recovery time and electrophysiologic tests has seldom been reported. Most of the studies considered that fENoG values <10% were associated with a poor outcome [17]. Cai el al. reported that fENoG values ≥ 33% were associated with a good recovery within half a year [18]. In our study, fENoG values above one-third determined by ROC curve analysis were associated with a favorable outcome and a shorter recovery time in patients receiving steroid treatments at the 6-month follow-up. In addition, patients with a delayed BR had a significantly better outcome than those with an absent BR, consistent with a previous study [7]. However, according to our analysis, fENoG was the most valuable prognostic factor, followed by the BR test.

The American Academy of Otolaryngology treatment guidelines highly recommend that oral steroids should be prescribed with 72 h of onset for Bell’s palsy [9]. Nevertheless, the optimal dosage and regimen of steroids remain unclear. Sullivan et al. reported that early treatment with prednisolone (at a dose of 25 mg twice daily) for 10 days improved the complete recovery rate [19]. Another study reported that early treatment with 60 mg of prednisolone for 5 days and then a reduction by 10 mg per day shortened the time to complete recovery [20]. In our study, a total of 382 (96.5%) patients received steroid treatment within 72 h. However, there were no significant differences between the high dose (≤0.5 mg/kg/day) and low dose (>0.5 mg/kg/day) or between the shorter regimen (≤7 days) and longer regimen (>7 days) in terms of the final outcome and recovery time.

There are some limitations in this study. First, this is a retrospective study, and we can only identify patients’ conditions based on medical records and electrophysiological reports. Second, most of the patients who received standard steroid treatment may have an increased overall favorable rate. Thus, the unfavorable outcome group was relatively small for an adequate comparison. Third, the timing of the electrophysiological test was performed 4–21 days after facial palsy onset. The bias of fENoG and BR tests may exist at different examination times. Finally, the study subjects came from a single medical center. The results may not represent the whole population. Nevertheless, our study provides useful information for predicting the outcome of Bell’s palsy after early steroid treatment. No similar studies had been reported in the population of Taiwan. Further large-scale prospective studies or meta-analysis should be performed to confirm these observations.

## 5. Conclusions

Our findings showed that a favorable outcome in Bell’s palsy was associated with a delayed BR and a large fENoG value. In addition, age younger than 60 years, delayed BR and fENoG values > 33% were associated with shorter recovery times. Therefore, this study may be helpful for clinicians taking a personalized medicine approach to identify patients with Bell’s palsy after adequate steroid treatment and to predict the future outcome according to related factors and electrophysiological tests.

## Figures and Tables

**Table 1:** Demographic and clinical variables of the patients with idiopathic Bell’s palsy.

Variable	All, *n* (%)	Favorable Outcome, *n* (%)	Unfavorable Outcome, *n* (%)	*p*-Value
Patients, *n*	396 (100)	354 (89.4)	42 (10.6)	
Age, years				0.354
<60	314 (79.3)	283 (79.9)	31 (73.8)	
≥60	82 (20.7)	71 (20.1)	11 (26.2)	
Sex				0.103
Female	179 (45.2)	165 (46.6)	14 (33.3)	
Male	217 (54.8)	189 (53.4)	28 (66.7)	
Affected site				0.279
Right	195 (50.8)	183 (51.7)	18 (42.9)	
Left	201 (49.2)	171 (48.3)	24 (57.1)	
Smoking				0.208
No	329 (83.1)	297 (83.9)	32 (76.2)	
Yes	67 (16.9)	57 (16.1)	10 (23.8)	
Alcohol drinking				0.226
No	352 (88.9)	317 (89.5)	35 (83.3)	
Yes	44 (11.1)	37 (10.5)	7 (16.7)	
Betel nut chewing				0.074
No	373 (94)	336 (94.9)	37 (88.1)	
Yes	23 (6)	18 (5.1)	5 (11.9)	
Diabetes				0.364
No	339 (85.6)	305 (86.2)	34 (81)	
Yes	57 (14.4)	49 (13.8)	8 (19)	
Hypertension				0.849
No	334 (84.3)	299 (84.5)	35 (83.3)	
Yes	62 (15.7)	55 (15.5)	7 (16.7)	
Hyperlipidemia				0.489
No	384 (97)	344 (97.2)	40 (95.2)	
Yes	12 (3)	10 (28.2)	2 (4.8)	
Blink reflex				0.0001 *
Delayed	149 (37.6)	145 (41)	4 (9.5)	
Absent	247 (62.4)	209 (59)	38 (90.5)	
Electroneurography				<0.0001 *
>33%	254 (64.1)	244 (68.9)	10 (23.8)	
≤33%	142 (35.9)	110 (31.1)	32 (76.2)	
Treatment				0.719
Supportive care	14 (3.5)	12 (3.4)	2 (4.8)	
Low-dose steroid ^a^	249 (62.9)	221 (62.4)	28 (66.7)	
High-dose steroid ^b^	133 (33.6)	121 (34.2)	12 (28.6)	

^a^ Low-dose steroid was defined as ≤0.5 mg/kg prednisolone/day. ^b^ High-dose steroid was defined as >0.5 mg/kg prednisolone/day. *: *p* < 0.05.

**Table 2:** Multivariable logistic regression analysis for favorable outcomes in patients with idiopathic Bell’s palsy (*n* = 396).

Variable	Odds Ratio (95% CI)
Age, years	
<60	1.23 (0.51 to 2.97)
≥60	1 (Reference)
Sex	
Female	1.53 (0.70 to 3.36)
Male	1 (Reference)
Smoking	
No	1.28 (0.35 to 3.92)
Yes	1 (Reference)
Alcohol drinking	
No	1.41 (0.34 to 5.88)
Yes	1 (Reference)
Betel nut chewing	
No	1.36 (0.28 to 6.55)
Yes	1 (Reference)
Diabetes	
No	1.21 (0.43 to 3.39)
Yes	1 (Reference)
Hypertension	
No	1 (Reference)
Yes	1.42 (0.44 to 4.54)
Hyperlipidemia	
No	2.52 (0.41 to 15.55)
Yes	1 (Reference)
Blink reflex	
Delayed	5.38 (1.82 to 15.90) *
Absent	1 (Reference)
Electroneurography	
>33%	6.67 (3.02 to 14.71) *
≤33%	1 (Reference)
Treatment	
Supportive care	1 (Reference)
Low-dose steroid ^a^	1.45 (0.22 to 9.72)
High-dose steroid ^b^	0.93 (0.13 to 6.60)
Duration	
≤7 days	2.41 (0.88 to 6.65)
>7 days	1 (Reference)

^a^ Low-dose steroid was defined as ≤0.5 mg/kg prednisolone/day. ^b^ High-dose steroid was defined as >0.5 mg/kg prednisolone/day. *: *p* < 0.05.

**Table 3:** Multivariable regression analysis for recovery time (days) in patients with idiopathic Bell’s palsy (*n* = 396).

Independent Variables	Coefficient	Standard Error	*t*-Value	*p*-Value
(Constant)	52.93			
Age	−8.02	3.40	−2.36	0.019 *
Blink reflex	−5.76	2.88	−2.00	0.046 *
Electroneurography	−14.84	2.90	−5.11	<0.0001 *

We defined age <60 as 1 and ≥60 as 0, delayed blink reflex as 1 and absent blink reflex as 0, and facial electroneurography value >33% as 1 and ≤33% as 0. *: *p* < 0.05.

## Data Availability

The data presented in this study are available on request from the corresponding author.
